# Unbiased Analysis of TCRα/β Chains at the Single-Cell Level in Human CD8^+^ T-Cell Subsets

**DOI:** 10.1371/journal.pone.0040386

**Published:** 2012-07-06

**Authors:** Xiaoming Sun, Masumichi Saito, Yoshinori Sato, Takayuki Chikata, Takuya Naruto, Tatsuhiko Ozawa, Eiji Kobayashi, Hiroyuki Kishi, Atsushi Muraguchi, Masafumi Takiguchi

**Affiliations:** 1 Center for AIDS Research, Kumamoto University, Honjo, Kumamoto, Japan; 2 Department of Immunology, Graduate School of Medicine and Pharmaceutical Sciences, University of Toyama, Toyama, Japan; MRC National Institute for Medical Research, United Kingdom

## Abstract

T-cell receptor (TCR) α/β chains are expressed on the surface of CD8^+^ T-cells and have been implicated in antigen recognition, activation, and proliferation. However, the methods for characterization of human TCRα/β chains have not been well established largely because of the complexity of their structures owing to the extensive genetic rearrangements that they undergo. Here we report the development of an integrated 5′-RACE and multiplex PCR method to amplify the full-length transcripts of TCRα/β at the single-cell level in human CD8^+^ subsets, including naive, central memory, early effector memory, late effector memory, and effector phenotypic cells. Using this method, with an approximately 47% and 62% of PCR success rate for TCRα and for TCRβ chains, respectively, we were able to analyze more than 1,000 reads of transcripts of each TCR chain. Our comprehensive analysis revealed the following: (1) chimeric rearrangements of TCRδ-α, (2) control of TCRα/β transcription with multiple transcriptional initiation sites, (3) altered utilization of TCRα/β chains in CD8^+^ subsets, and (4) strong association between the clonal size of TCRα/β chains and the effector phenotype of CD8^+^ T-cells. Based on these findings, we conclude that our method is a useful tool to identify the dynamics of the TCRα/β repertoire, and provides new insights into the study of human TCRα/β chains.

## Introduction

CD8^+^ T cells play an important role in adaptive immunity against virus-infected cells and tumor cells [1]–[3]. In the primary antigen response, naive CD8^+^ T cells are activated in secondary lymph nodes and consequently undergo clonal expansion and differentiation into effector and memory CD8^+^ cells that sequentially circulate in the periphery in vivo [4], [5]. Effector CD8^+^ T cells have direct effector functions such as cytotoxic activity and cytokine production in response to the target cells, whereas memory CD8^+^ T cells do not show these functions, but have the ability to proliferate and secrete large amounts of cytokines when the cells are stimulated by antigens [6].

T-cell receptor (TCR)α/β chains are heterodimeric membrane proteins expressed on the surface of CD8^+^ T-cells, and they contribute to direct recognition of antigen peptide presented on the major histocompatibility complex (MHC) in the target cells [7], [8]. The specificity of antigen recognition for diverse peptide-MHC (pMHC) complexes depends on the 3 complementarity determining regions (CDRs) of both TCRα and TCRβ chains. CDR1 and CDR2 are encoded by the germline sequences and mainly used for the binding to the MHC, whereas CDR3 is known to be the highly polymorphic and the principal antigen recognition site created by extensive genomic rearrangement occurring among variable (V), diversity (D), and joining (J) segments. The diversity of CDR3 is further generated by the deletion and insertion of nucleotides within the junction of V-J and V-D-J in TCRα and TCRβ chains, respectively [9]–[11].

Methods to characterize the diversity and clonality of the TCRα/β repertoire have been previously described and remarkably improved by the development of recent technologies such as TCR spectratyping [12]–[14] and deep sequencing [15]–[18]. However, most approaches have focused on the characterization of a single TCRβ chain without consideration of the TCRα/β pairs that determine the actual TCR diversity and clonotype. There are some methods that have been described for the analysis of paired TCRα/β chain transcripts from single cells, but these methods are limited to activated human T-cells *in vitro* or antigen-specific mouse T-cells *ex vivo* [19]–[21].

The TCR amplification methods are basically categorized into 2 groups based on the utilization of the 5′rapid amplification of cDNA end (RACE) method [22] or the multiplex PCR method [23]. The 5′-RACE method can exclude potential bias and provide full-length TCRα/β transcripts that are useful for sequential study such as that of TCRα/β transduction, which analyzes the specificity of an antigen. However, the specificity and efficiency of PCR amplification in the 5′-RACE method are low, especially when there is contamination by short fragments created by mRNA degradation or incomplete cDNA synthesis in the reverse transcription process. In contrast, the multiplex PCR method gives better specificity and efficiency of PCR than the 5′-RACE method but does not provide the full length of TCRα/β transcripts. In addition, it potentially has bias because of multiple primers designed for each variable segment of TCRα/β chains.

Here, we report an unbiased method developed by the integration of 5′RACE and multiple PCR methods for amplification of the full-length and paired TCRα/β chain transcripts at the single cell level in human CD8^+^ T-cell subsets. This method has wide applications and has allowed us to demonstrate chimeric rearrangements in TCRα/β chains, regulation of TCRα/β chain expression with multiple transcriptional initiation sites, and dynamics of the TCRα/β repertoire among different subsets of human CD8^+^ T cells.

## Results

### Amplification of full-length TCRα/β chain transcripts from single CD8^+^ T-cells

**Figure 1:**
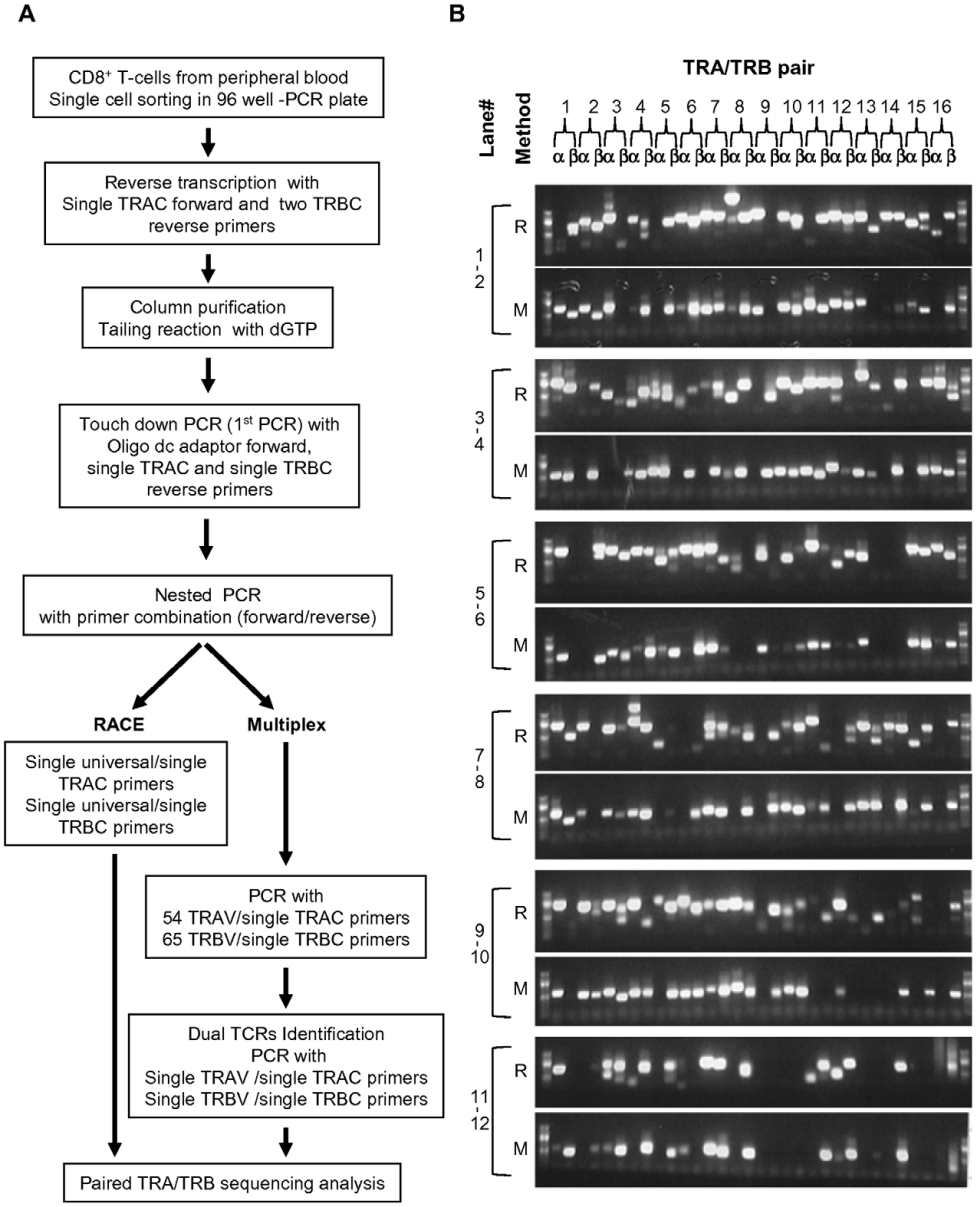
Amplification of TCRα/β chains from single CD8^+^ T-cells. (A) Experimental strategy for amplifying and sequencing TCRα/β chains by integrated 5′-RACE (RACE) and multiplex PCR (Multiplex). (B) Visualization of TCRα/β chain transcripts by agarose gel electrophoresis. The results were obtained from 96 cells in the naive subset in donor 1 and are shown as a representative result. R: 5′-RACE, M: mutitiplex PCR.

**Figure 2:**
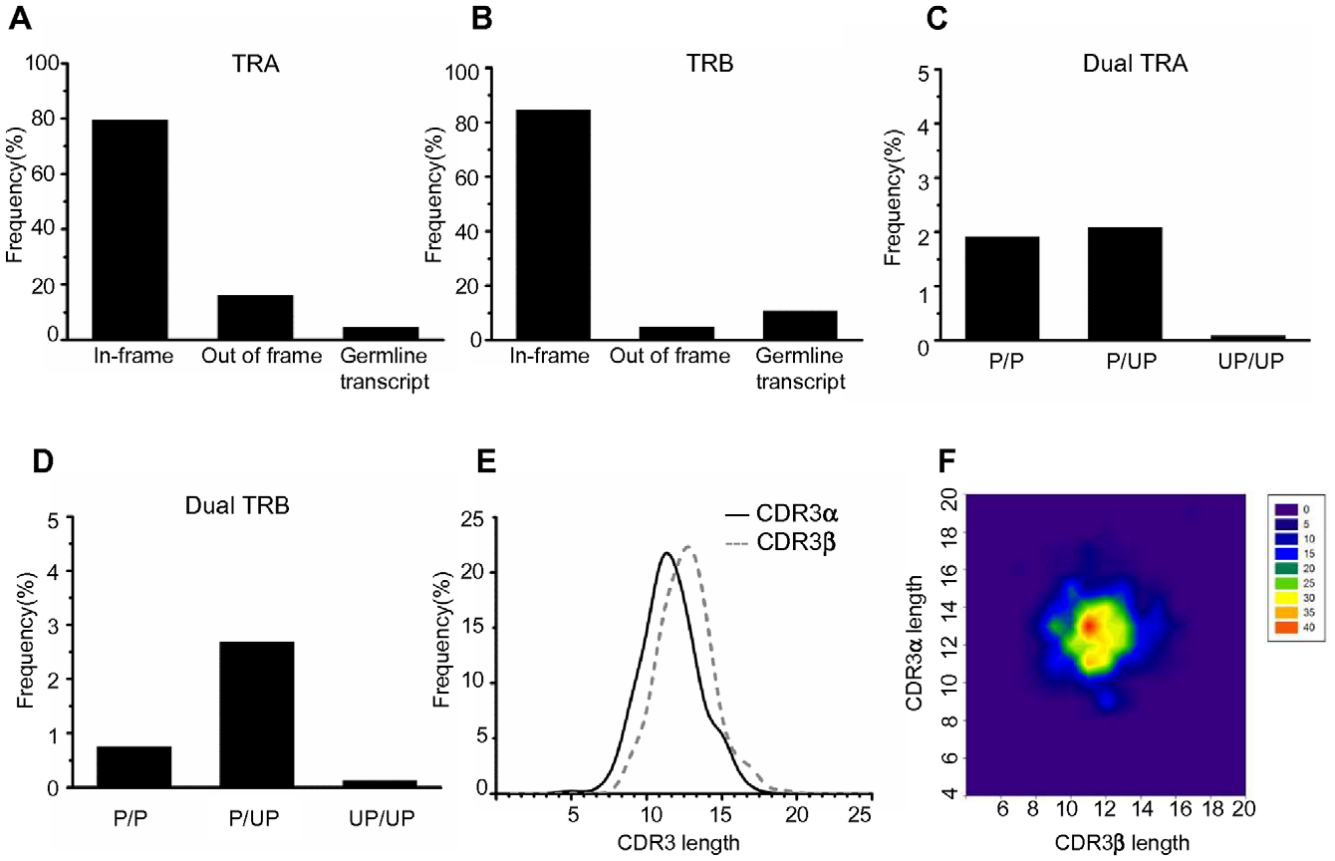
Characterization of **TCRα/β-chain transcripts expressed in CD8^+^ T-cells.** For characterization of TCRα/β chain transcripts, 1,250 and 1,661 sequence reads were used for TCRα and TCRβ chains, respectively. (A) Frequency of in-frame, out-of-frame, and germline transcripts for TCRα chains. Out-of-frame transcripts represent a sequence that contains either a stop codon or a frame-shift, whereas germline transcripts are defined by the absence of a variable segment and evidence that the transcript was started from a genomic region located in the upstream of either a joining or diversity segment. (B) Frequency of in-frame, out-of-frame, and germline transcripts for TCRβ chains. (C) Frequency of dual TCRα chain transcripts. The samples that had 2 transcripts were defined as dual TCRs. P: productive TCRs representing in-frame transcripts. UP: unproductive TCRs representing out-of-frame transcripts. (D) Frequency of dual TCRβ-chain transcripts. (E) Size distribution of CDR3α and CDR3β lengths. Samples representing productive TCRs (1,011 and 1,444 reads for TCRα and TCRβ chains, respectively) were extracted from the whole data set. The CDR3 lengths were identified by the IMGT/V-Quest tool at the amino acid (aa) level. (F) Paired analysis of CDR3α and CDR3β lengths. By use of 736 samples, the most frequent pair was identified at the position intersecting 11 and 13 aa of CDR3α and CDR3β lengths, respectively, as shown in red.

We applied the integrated 5′-RACE and multiplex PCR method for amplification of the full length of both TCRα and TCRβ chain transcripts from single cells in CD8^+^ T-cell subsets including naive (CD27^high^CD28^+^CD45RA^+^CCR7^+^), central memory (CD27^+^CD28^+^CD45RA^-^CCR7^+^), early effector memory (CD27^+^CD28^+^CD45RA^−^CCR7^−^), late effector memory (CD27^low^CD28^−^CD45RA^+/−^CCR7^−^), and effector (CD27^−^CD28^−^CD45RA^+/−^CCR7^−^) phenotypic populations obtained from the peripheral blood of 3 unrelated donors (Table 1) [24]. The PCR amplifications were successfully performed (Figure 1), and the overall PCR success rate was approximately 47% for TCRα and 62% for TCRβ chains. This result is consistent with a previous report indicating that the PCR success rate for TCRβ chains is slightly better than that for TCRα chains in mice [19]. Although the results of PCR amplification given by 5′-RACE and multiplex PCR methods were almost identical, as shown in Figure 1B, a subset of TCRα/β chain transcripts appeared once from either 5′-RACE or and multiplex PCR method. These results indicate that the integration of 5′-RACE and multiplex PCR methods could increase the PCR success rate. Indeed, we selected 1250 samples for analysis (974 samples from 5′RACE and 276 samples from Multiplex PCR method) for TCRα chain and 1661 samples (1075 samples from 5′RACE and 586 samples from Multiplex PCR method) for TCRβ chain (Table S1). Form these data, we found that approximately 80% of TCRα and 87% of TCRβ chains were in-frame and that approximately 16% of TCRα chains and 6% of TCRβ chains were out of frame. Among the chains, 3.4% of TCRα ones and 5.8% of TCRβ ones were germline transcripts that lacked the variable segments and showed unproductive TCRs (Figure 2A and 2B) [25], [26]. All germline transcripts were detected by 5′ RACE, but not by the multiplex PCR method. A fraction of samples failed to identify the TCR because of unreadable sequences, which may indicate the existence of dual TCRs. Therefore, we performed PCR with a single gene-specific primer designed for the middle of the variable region in the TCRα/β chains, and the sequence analysis revealed that productive dual TCRα and TCRβ chains were expressed in approximately 1.9% and 0.8% of CD8^+^ T-cells, respectively (Figure 2C and 2D). Approximately 14% of CD8^+^ T-cells had only unproductive TCRα (Fig2A and C), though productive TCRβ chain was detected in a half of these cells. We did not clarify whether these cells express only TCRβ chain because success rate to detect TCRα chain by this method is approximately 50%. Furthermore, consistent with a previous report [19], the most frequent dual TCRα and TCRβ were observed with a combination of productive TCR and unproductive TCR, the latter of which had either a stop codon or frame-shift.

**Table 1:** Success rate of single-cell PCR for TCRα/β chains in CD8^+^ T-cell subsets.

	Donor 1					Donor 2					Donor 3				
Age	26					34					29				
Sex	Male					Male					Male				
HLA type	A*31:01/A*31:01/B*40:06/B*56:01/C*04:01/C*08:01					A*01:01/A*24:02/B*37:01/B*40:02/C*03:03/C*06:02					A*02:01/A*24:02/B*51:01/B*52:01/C*12:02/C*04:01				
CD8^+^T-cell subset	N	CM	EEM	LEM	E	N	CM	EEM	LEM	E	N	CM	EEM	LEM	E
Population (%)	46.3	1.64	17.0	2.12	5.97	67.9	1.17	9.14	1.06	2.87	47.1	0.74	8.47	5.24	19.9
Total # of cells analyzed for TRA	92	77	74	82	67	90	75	75	72	83	90	74	81	87	84
PCR success rate for TRA (%)	63.9	68.8	61.7	56.9	39.9	45.8	39.1	19.5	37.5	43.2	62.5	38.5	42.2	45.3	43.8
Total # of cells analyzed for TRB	119	90	86	92	98	118	132	106	105	102	115	108	109	111	113
PCR success rate for TRB (%)	82.6	80.4	71.7	63.9	58.3	61.5	68.8	27.6	54.7	53.1	79.9	56.3	56.8	57.8	58.9

Since CDR3 is the most diverse region, created by the deletion and insertion of nucleotides within the junction of V-J and V-D-J in TCRα and TCRβ chains, respectively, we analyzed the distribution of CDR3 length at the amino acid (aa) level in TCRα and TCRβ chains by using samples showing only productive TCR defined by the in-frame sequence (1,011 and 1,444 reads for TCRα and TCRβ chains, respectively). The analysis demonstrated that the length of CDR3α ranged from 4 to 18 aa, whereas the length of CDR3β was slightly longer, ranging from 5 to 20 aa (Figure 2E). Further analysis of paired CDR3α and CDR3β lengths with 736 samples identified the most frequent pair at the position intersecting 11 and 13 aa of CDR3α and CDR3β lengths, respectively (Figure 2F). These results suggest that human TCRα/β chains may have a preferential combination of CDR3α and CDR3β lengths for recognition of diverse pMHC complexes.

### Identification of transcriptional initiation sites (TISs) in genes of TCRα/β chains

**Figure 3:**
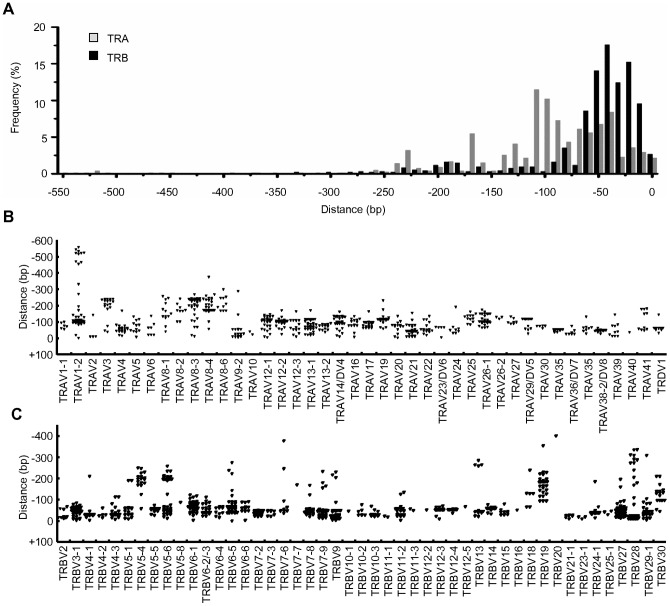
Identification of transcription initiation sites (TISs) in TCRα/β chain. (A) Distribution of TISs in TCRα/β chains. Samples containing a translation initiation site (773 and 930 reads for TCRα and TCRβ chains, respectively) were used for the analysis, and the initiation site was defined as zero. (B) Distribution of TISs in TRAVs of TCRα chains. Using the same data set, TISs were determined for individual TRAVs. (C) Distribution of TISs in TRBVs of TCRβ chains. A single dot represents a position of TIS obtained from a single sequence read.

Our integrated method included 5′-RACE, which is able to amplify the full length of transcripts and has been used to identify the TIS of genes. Therefore, we examined whether our sequence data obtained from single cells also had the power to identify the TISs of TCRα/β chains. At first we collected samples that showed the presence of a translational initiation site in TCRα/β chains, and then the sequences were aligned with the genomic sequences to measure the length of the 5′-UTR (5′-untranslated region) that eventually informs the position of the TCRα/β TISs. With 773 and 930 reads for TCRα and TCRβ chains, respectively, the distribution of TISs in the genes of TCRα/β chains showed that the TISs of the TCRβ chain accumulated around 40-bp downstream of the translational initiation sites but that those of the TCRα chain were concentrated in 2 locations, i.e., 40 bp and 110 bp downstream of the translational initiation sites (Figure 3A). The TISs in individual TCRα/β variable segment (TRAV and TRBV) were also analyzed, and the results showed that subsets of TRAV and TRBV transcripts started from more than 2 positions (Figure 3B and 3C), suggesting that there are multiple binding sites for transcription factors that tightly control the expression of TCRα/β chains restricted in T lymphocytes [27], [28].

### Usage of TCRα/β variable segments in human CD8^+^ T-cell subset

**Figure 4:**
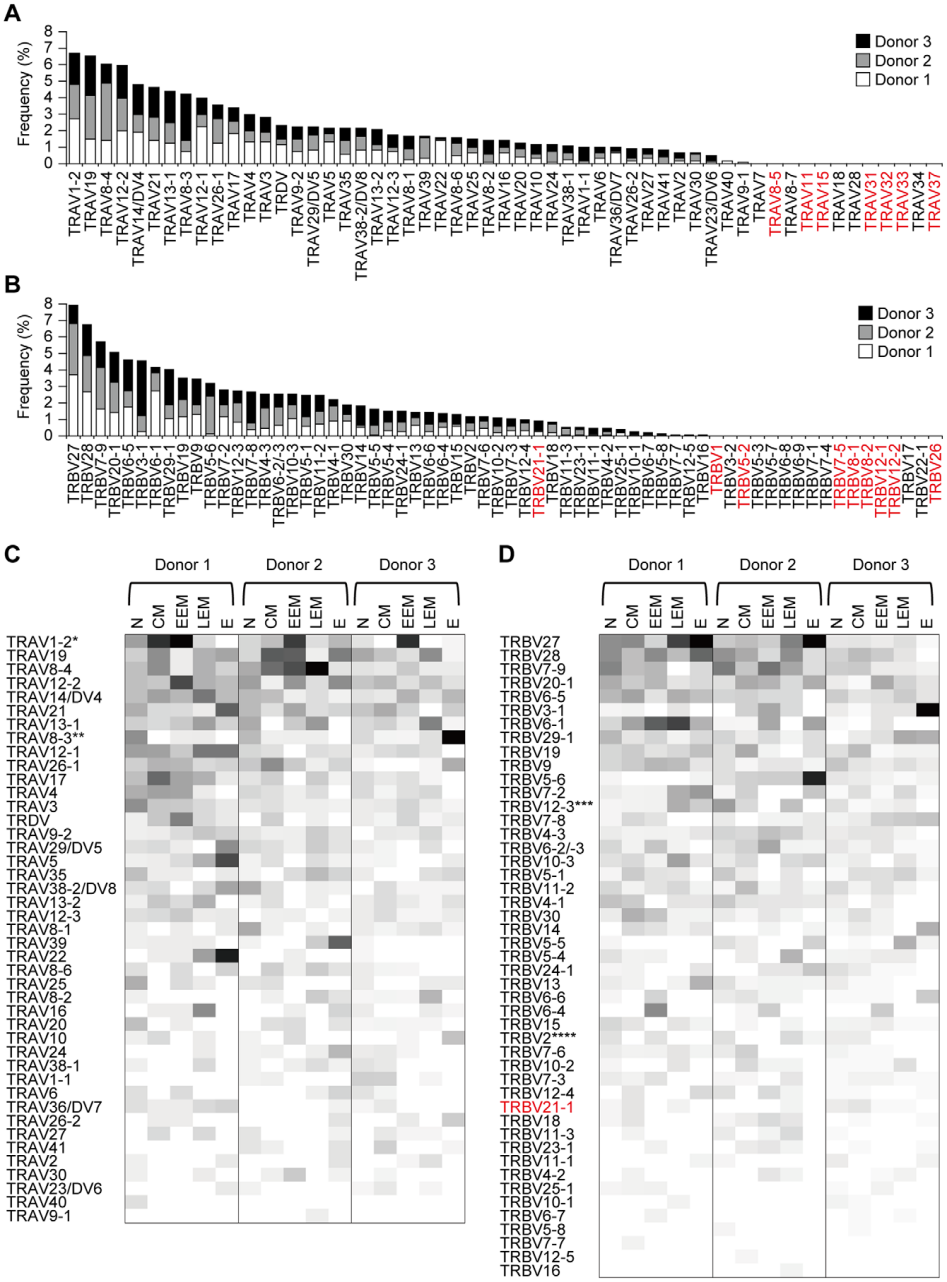
TRAV and TRBV usage in CD8+ T-cell subsets among 3 unrelated donors. (A) Frequency of TRAV usage in the 3 donors. (B) Frequency of TRBV usage in the 3 donors. (C-D) TRAV (C) and TRBV (D) usage in CD8^+^ T-cell subsets among the 3 donors. The same data set used for TIS analysis was used for all analysis of TRAV and TRBV usage. The frequency is proportional to the density of colors: white (low) to black (high). The pseudogenes of TRAV and TRBV are shown in red. N: naive, CM: central memory, EEM: early effector memory, LEM: late effector memory, E: effector. Statistical analysis was performed using the χ2 and Student' s t test. *: *P*<0.05, EEM>N, EEM>LEM, EEM>E, **: *P*<0.05, N>CM, N>EEM, N>LEM, ***: *P*<0.05, LEM>EEM, ****: *P*<0.05, N>E.

To analyze the usage of TRAVs and TRBVs in human CD8^+^ T-cell subset, we extracted samples including the variable segments but not those identified as germline transcripts from the whole sequence data set. The assembly of the samples obtained from 1,207 and 1,540 reads for TCRα and TCRβ chains, respectively (Table S2), demonstrated that 42 out of 54 TRAVs and 47 out of 64 TRBVs were detected in the CD8^+^ T-cell subset with different frequencies but that all pseudogenes except TRBV21-1 were not detectable (Figure 4A and 4B). The IMGT data base together with a previous report [15] has defined TRBV21-1 as a pseudogene based on the fact that it has a frame-shift in the leader sequence, but about 30% of our samples carrying the TRBV21-1 rearrangement appeared as productive TCRβ chains encoding CDR1, CDR2, and CDR3 domains with in-frame sequences in the entire transcript (data not shown), suggesting that TRBV21-1 may function and be required for antigen recognition *in vivo.* There were several TRBV segments that were not detected in this analysis. Interestingly, we found TRDV-TRAJ rearrangement in a substantial number of TCRα transcripts (Figure S1). These results, along with the finding of TRBV21-1 utilization, define the requirement of the 5′-RACE method and the limitation of the multiplex PCR method if multiple primers are designed for variable segments in TCRα/β chains.

Using the same data set, we next analyzed the usage of TRAVs and TRBVs in each CD8^+^ subset among 3 unrelated donors (Figure 4C and 4D). The results indicated that the usage of TRAV1-2 in all of the donors was significantly higher in the early effector memory cells than in other 3 subsets and that the usage of TRAV8-3 was significantly higher in naïve subset than in other 3 subsets (Figure 4C). The usage of TRBV12-3 was significantly lower in early effector memory subset than in late effector memory subset while that of TRBV2 was significantly higher in naïve subset than in effector subset (Figure 4D). Furthermore, the detailed analysis of TRAV1-2 showed that most TRAV1-2 had rearranged with TRAJ33 and that CDR3α was highly conserved among samples and also among donors (Figure S2 and Table S3). In addition, the paired TCRα/β analysis showed that the TRBV6 subgroup was preferentially used for the pairing with TRAV1-2-TRAJ33 rearranged within the TCRα chain.

### Usage of joining and diversity segments of TCRα/β chains in human CD8^+^ T-cell subset

**Figure 5:**
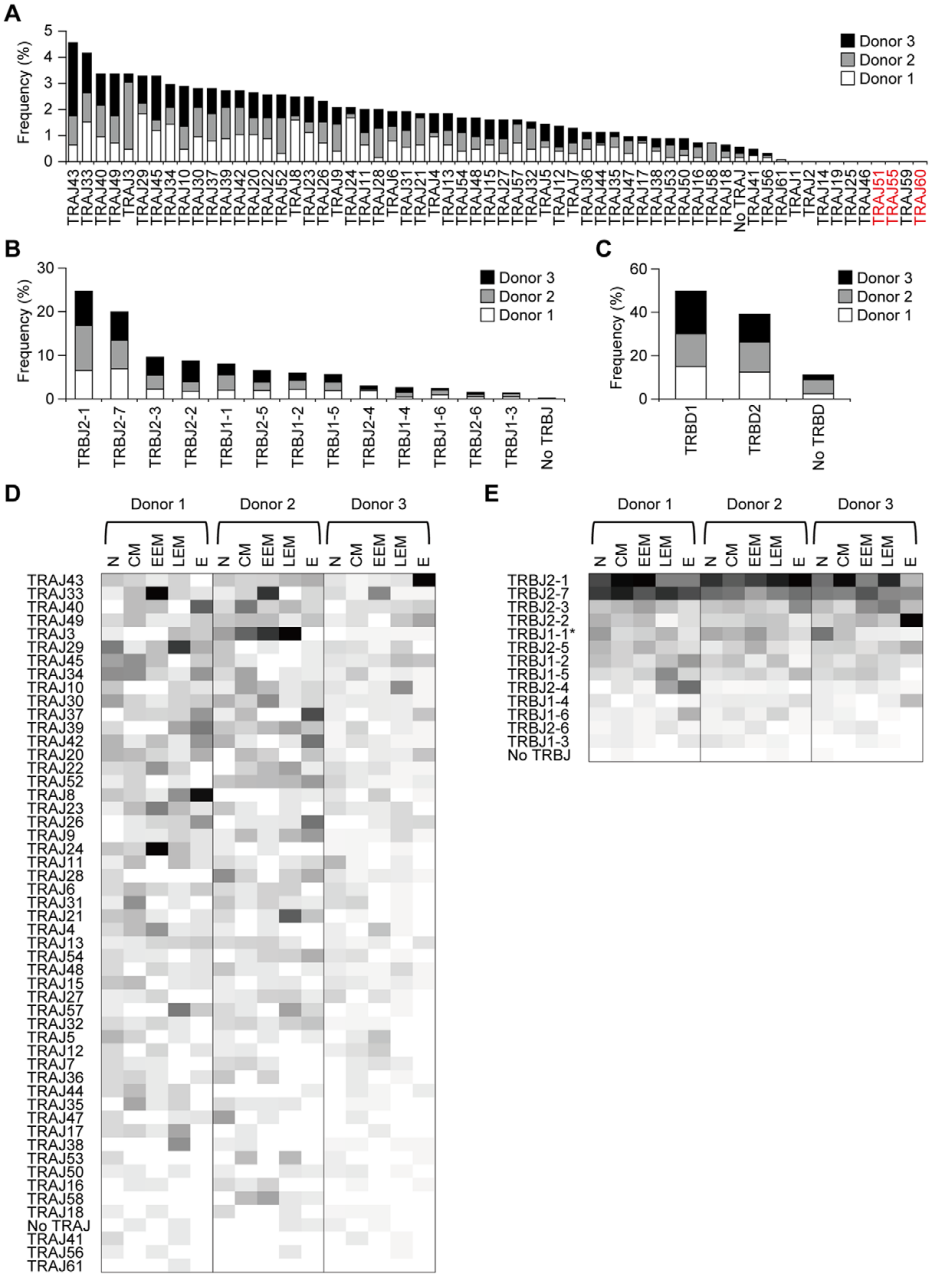
TRAJ, TRBJ, and TRBD usage in CD8^+^ T-cell subsets among the 3 unrelated donors. (A) Frequency of TRAJ usage in the 3 donors. (B) Frequency of TRBJ usage in the same donors. (C) Frequency of TRBD usage in the 3 donors. (D) TRAJ usage in CD8^+^ T-cell subsets among the 3 donors. (E) TRBJ usage in CD8^+^ T-cell subsets among the same donors. The frequency is proportional to the density of colors from white (low) to black (high). The pseudotypes of TRAJ and TRBJ are shown in red. Statistical analysis was performed using the χ2 and Student's t test. *: *P*<0.05, N>EEM, N>E.

The analysis of usage of joining and diversity segments (TRAJ, TRBJ, and TRBD) was performed on the same data set as used for the TRAV and TRBV usage analysis. The result showed that 51 out of 61 TRAJs and 13 out of 14 TRBJs in TCRα/β chains were detected with different frequencies in the CD8^+^ T-cell subset of the 3 donors (Figure 5A and 5B). Consistent with the observation made by TRAV and TRBV usage analysis, we found that several joining segments defined as pseudogenes were not detectable in any of the CD8^+^ T-cell subsets. There was no significant difference in the usage of TRBD1 and TRBD2 among the 3 donors (Figure 5C). We observed that a subset of TCRα/β chain transcripts lacked TRAJ, TRBJ or TRBD. This result suggests that the transcripts lacking TRAJ and TRBJ may have been due to splicing errors and that the lack of TRBD may have been a consequence of deletion of its nucleotides during the process of V-D-J recombination.

To analyze the usage of TRAJ and TRBJ in each CD8^+^ subset among the 3 unrelated donors, we used the same data set. The analysis demonstrated that the usage of TRBJ1-1 was significantly higher in naïve subset than in early effector memory and effector subsets (Figure 5E).

### Identity and clonotype of TCRα/β chains in human CD8^+^ T-cell subset

**Figure 6:**
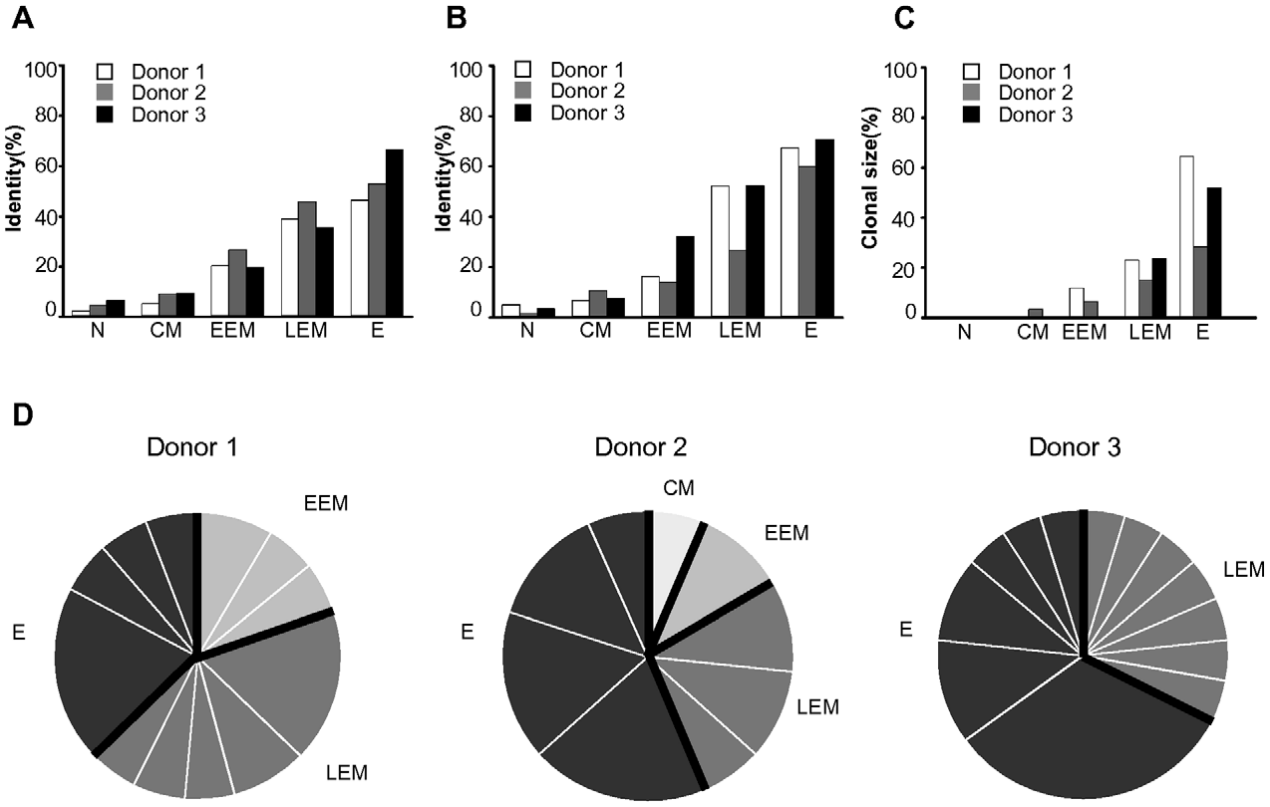
Identity and clonotype of TCRα/β chains in CD8^+^ T-cell subsets among the 3 unrelated donors. A data set not including samples showing germline transcripts was used for the analysis (1,207 and 1,540 reads for TCRα and TCRβ chains, respectively). (A) Frequency of TCRα chain identity in CD8^+^ T-cell subsets. The identity was defined as more than 2 appearances of samples with the identical usage of TRAV, TRAJ, and CDR3α. (B) Frequency of identical TCRα and β chains in CD8^+^ T-cell subsets. The identity was defined as more than 2 appearances of samples with the identical usage of TRBV, TRBD, TRBJ, and CDR3β. (C) Frequency of paired TCRα/β clonotype in CD8^+^ T-cell subsets. The percentage was measured by using a data set obtained from 901 paired TCRα/β sequence reads. The clonotype was defined by the more than 2 appearances of samples showing identical TCRα/β chains. (D) Proportion of TCRα/β clonotypes in CD8^+^ T-cell subsets among the 3 unrelated donors. The TCRα/β clonotypes identified in “C” were discriminated by type and proportion (see Table S4).

Given the fact that antigen-experienced CD8^+^ T-cells clonally proliferate after activation and sequentially differentiate into memory phenotypic CD8^+^ T-cells that circulate in the periphery *in vivo*, it is possible that the identity and clonotype of TCRα/β chains may be in different proportions among CD8^+^ T-cell subsets. To examine this possibility, we at first analyzed the identity of TCRα and TCRβ chains individually in each CD8^+^ T-cell subset by using the same data set as used for the TRAV and TRBV usage analysis. The results showed that the identity of both TCRα and TCRβ chains had gradually increased from naive cells to effector cells, which had been phenotypically classified beforehand (Figure 6A and 6B) [24]. In addition, approximately 60% of effector cells showed identical TCRα/β chains once at least, suggesting the occurrence of clonal expansion *in vivo*. Using a data set that provided the paired TCRα/β chain transcripts (901 paired sequence reads), we next analyzed the clonotype of TCRα and TCRβ chains by satisfying the following requirements for the different samples in each CD8^+^ T-cell subset of the 3 donors: (1) identical usage of variable, diversity, and joining segments in TCRα/β chains and (2) perfect matching of CDR3 length and sequence at the amino acid level in TCRα/β chains. The analysis revealed that the largest proportion of clonotype was the effector subset, with none or a smaller proportion of it as naive, central memory, early effector memory or late effector memory subset (Figure 6C). This result shows that the clonal size of CD8^+^ T-cells was associated with the effector phenotype, as had been previously described and classified by the degree of expression of 3 effector molecules [24]. Further analysis demonstrated that the number, type, and size of TCRα/β clonotypes were different among the donors having different HLA-types with the exception of the HLA-A*24:02 type in donor 2 and donor 3 (Figure 6D and Table S4), suggesting that the cells showing the TCRα/β clonotype were clonally expanded after activation by the recognition of different pMHC complexes *in vivo*.

## Discussion

The methods for TCR amplification have been previously reported and continuously improved by the development of recent technology [12]–[16], [18]–[21]. However, we were unable to find any suitable method for our analysis based on our requirements, such as the amplification of full-length and paired TCRα/β chains from human CD8^+^ T-cells at the single cell level with high PCR success rate and no potential bias. Hence, we newly developed an integrated method originating from 5′-RACE and multiplex PCR methods. We found that our method successfully worked across human CD8^+^ T-cell subsets obtained from 3 unrelated individuals without a reduction in PCR success rate compared with that obtained with methods previously described [19], [21]. In the comprehensive TCRα/β analysis, the necessity of our integrated method was emphasized by the following evidence: 1) a subset of TCRα/β chain transcripts was amplified with either the 5′-RACE or multiplex PCR method, 2) the 5′-RACE, but not multiplex PCR, method could amplify germline transcripts and transcripts carrying the chimeric rearrangements of TCRδ-TCRα 3) the combination of multiplex PCR and sequential PCR with single TRAV or TRBV primers identified the existence of dual TCR expression in single CD8^+^ T-cells. In light of this evidence, we expect that the full-length of paired TCRα/β chain transcripts amplified by the 5′-RACE method will be useful for not only the identification of transcription initiation sites, but also for simplifying the strategy of TCRα/β tranduction to confirm the pairing and potential antigen recognition through the direct cloning into lenti-virus expression vectors, as described previously [29].

The regulation of mono- or bi-allelic expression at TCR and immmunoglobulin (Ig) loci is known to involve changes in chromatin structure, methylation, and replication timing in 2 identical alleles [30]–[33]. In our data set, about 80% of CD8^+^ T-cells showed mono-allelic expression of TCRα and TCRβ chains, with expression of dual TCRα and dual TCRβ in approximately 3% of them. However, a previous study demonstrated that dual TCRα expression, but not dual TCRβ expression, was detected in 10∼20% of influenza-specific peripheral CD8^+^ T-cells [19], suggesting that the difference in the frequency of dual TCR may depend on the type of cells targeted. Indeed, we found that 9.2% of early effector cells from the donor 1 expressed dual TCRα chains with single TCRβ chains and that there was no association between CD8^+^ T-cell subsets and the frequency of dual TCRs among the 3 unrelated donors.

The germilne transcription coding for unproductive TCR and Ig composed of D-J-C or J-C segments has been found to occur before the V(D)J rearrangement, and is thought to be driven by a developmental stage-specific promoter that should be activated in immature cells [25], [26], [34]. A striking observation in our data was the expression of germline transcripts in peripheral CD8^+^ T-cells, where we found that most of these transcripts were detected together with a productive TCR transcript in a subset of CD8^+^ T-cells. This finding, together with the result that approximately 80% of the CD8^+^ T-cells analyzed in this study expressed a single TCR support the idea that these germline transcripts were expressed in immature T-cells by biallelic activation at D or J locus, but that only 1 of the 2 alleles was inactivated in mature T-cells after V-(D)-J rearrangement through methylation and the changes in chromatin structure. Some of the mature T-cells analyzed escaped in this manner.

TISs have been investigated in murine and human TCRβ chains, with the finding that the positions fall into a range of 19–40 bp and 26 bp upstream of the translation initiation site in most murine TRBVs and human TRBV7-2 (Vβ6.7), respectively [27], [28]. These results are consistent with ours in that the positions of TISs were located quite close to the translational initiation site. However, our finding that there were multiple transcription sites in a subset of TRBVs as well as TRAVs, and the fact that the promoters located in each TRBV did not have uniform transcriptional activity, suggests that the regulatory mechanism of TCRα/β expression may be more complicated and that these events may be implicated in the usage of variable segments in TCRα/β chains in CD8^+^ T-cell subsets.

Chimeric TCRs created by the rearrangement of Vδ-Jα has been found in peripheral T-cells of mice and humans, and the heterodimerization of chimeric δα TCR and TCRβ chains can be expressed on the surface of CD8^+^ T-cells and they recognize antigen presented by antigen-presenting cells [35]–[37]. We also found that a subset of CD8^+^ T-cells expressed the chimeric δα TCR chain together with the TCRβ chain. We are not able to know the function of the chimeric δα TCR chain, owing to the limitations of our technology, but the finding of the clonotype showing the identical pair of chimeric δα TCR chain and TCRβ chains in the early effector memory subset suggests that these cells had some function in response to antigen stimulation *in vivo*. These findings suggest that the diversity of human TCRα/β genes may be greater than previously estimated [14].

The expression of TCRα/β chains varied among CD8^+^ T-cell subsets in the 3 unrelated donors having different HLA-types (except for HLA-A*24:02 type in donor 2 and donor 3), but a finding that about 10∼20% of early effector memory cells in all 3 unrelated donors expressed a particular type of TCRα chain carrying a rearrangement of TRAV1-2 and TRAJ33 is interesting from an immunological point of view. Although further study with an increased number of donors will be necessary, a detailed analysis showing the highly conserved CDR3α in rearrangements of TRAV1-2 and TRAJ33 and the preferential usage of the TRBV6 subgroup as its partner may suggest that these early effector memory cells are distinct from the other subsets and recognize various pMHC complexes with some similarity at the level of protein conformation.

In summary, we described herein an unbiased method for amplification of paired TCRα/β chains at the single-cell level. We believe that our method is novel and has the potential for a wide range of applications. Indeed, the application of the method for the characterization of TCRα/β chains in CD8^+^ T-cell subsets could provide the first evidence that the proportion of TCRα/β identity and clonotypes is associated with the effector function of CD8^+^ T-cells. We expect that our method using phenotypic classification of CD8^+^ and CD4^+^ T-cells will be a useful tool to identify the dynamics of TCRα/β genes in patients with various infectious diseases or tumors and may contribute to the immunotherapy of them.

## Materials and Methods

### Sample preparation

Human peripheral blood mononuclear cells (PBMCs) were prepared from heparinized peripheral blood from 3 unrelated donors (Table 1), using Ficoll-Paque PLUS (GE Healthcare, Uppsala, Sweden), and stored in liquid nitrogen. Before use, the PBMCs were rested overnight in culture media (RPMI 1640 supplemented with 10% FCS, 100 U/ml MEM-NEAA, 100 U/ml sodium pyruvate, and 200 U/ml recombinant human IL-2). This study was approved by the Kumamoto University Ethical Committee, and written informed consent was obtained from all participants.

### Cell staining and single-cell sorting

Surface staining of PBMCs and classification into CD8^+^ T-cell subsets were performed as described previously [24]. Single cells sorted from each of the CD8^+^ T-cell subsets by use of a FACSAria equipped with 405-, 488-, and 633-nm lasers and FACSDiva acquisition software (BD Biosciences) were plated into a 96-well plate containing cell lysis buffer (see below).

### Single-cell RT-PCR with integrated 5′-RACE and multiplex PCR

The sorted single cells were plated into each well of a 96-well plate with 2 μl of cell lysis buffer containing 1.5 μl of resuspension buffer (Invitrogen), 0.1 μl of lysis enhancer solution (Invitrogen), 0.03 μl of 25 mM dNTPs, 0.1 μl of 40,000U/ml RNase inhibitor, and 0.22 μl of a primer mixture (10 μM concentration of each of hTCR-CA-R2.2, hTCR-CB1-R3.2, and hTCR-CB2-R3 primer) (Table S5). The cells were incubated at 75°C for 10 min and then put on ice immediately. cDNA was synthesized directly from cell lysates by using 6.0 μl of reverse-transcription (RT) solution consisting of 1.2 μl of 5x 1^st^- strand DNA buffer (Invitrogen), 0.19 μl of 0.1M DTT (Invitrogen), 0.19 μl of RNase inhibitor (NEB), 0.19 μl of SuperScriptIII Reverse Transcriptase (Invitrogen), and 2.33 μl of DEPC-treated H_2_O (Invitrogen). RT reactions were performed at 54°C for 60 minutes followed by incubation at 85°C for 5 minutes. Template RNA was digested with 1U of RNase H (Invitrogen) at 37°C for 20 minutes. Extra primers and dNTPs were removed from RT samples by use of a Zymo-Spin™ I-96 Plate (ZYMO Research) according to the manufacturer's instructions. The purified cDNA was incubated at 94°C for 3 minutes and subsequently on ice for at least 2 minutes, and then the tailing of the cDNA was performed with 2 μl of tailing solution consisting of 0.15 μl of 10 mM dGTP (Promega), 0.15 μl of 1M P-K buffer (1M K_2_HPO_4_, 1M KH_2_PO_4_; pH7.0), 0.48 μl of 25 mM MgCl_2_ (Promega)_,_ 40 U/μl TdT (Roche), and 1.12 μl of water. The tailing was performed at 37°C for 60 minutes followed by incubation at 65°C for 10 minutes. For amplification of TCRα- and TCRβ-chain transcripts, touch-down PCR (first-round PCR) was performed in 25 μl of 2x primeSTAR GC buffer (TaKaRa), 4 μl of 2.5 mM dNTPs (TaKaRa), 1 μl of 10 μM Oligo-dc-adaptor2, 1 μl of 10 μM hTCR-CA-R7, 1 μl of 10 μM hTCR-CB1-R9, 0.625 U of PrimeSTAR (TaKaRa), and 5.75 μl of water, using the following conditions: 1) 96°C for 2 minutes, 2) 3 cycles of 96°C for 15 seconds and 72°C for 2 minutes, 3) 3 cycles of 96°C for 15 seconds, 69°C for 15 seconds, and 72°C for 1.5 minutes, 4) 3 cycles of 96°C for 15 seconds, 66°C for 15 seconds, and 72°C for 1.5 minutes, 5) 26 cycles of 96°C for 15 seconds, 63°C for 15 seconds, and 72°C for 1.5 minutes. Using 1 μl of a 1∶20 dilution of the first-round PCR reactions, a nested second PCR was performed in a 20 μl reaction mixture consisting of 10 μl of 2x PrimeSTAR GC buffer (TaKaRa), 1.6 μl of 2.5 mM dNTPs (TaKaRa), 0.1 μl of 2.5 U/μl PrimeSTAR (TaKaRa), 6.66 μl of water, 0.32 μl of 10 μM AP2, and 0.32 μl of 10 μM reverse primer corresponding to the TCR constant region (hTCR-CA-R9 for TCRα chain and hTCR-CB1-R6 for TCRβ chain). The PCR conditions were as follow: 1) 96°C for 2 minutes, 2) 35 cycles of 96°C for 15 seconds, 58°C for 30 seconds, and 72°C for 1 minute, and 3) 72°C for 3 minutes. Multiplex PCR was also performed with 2.5 μl of the first-round PCR product, 10 μl of Taq colorless buffer (Promega), 3 μl of 25 mM MgCl_2_ (Promega), 4 μl 2.5 mM dNTPs (Promega), 50 U of Taq DNA polymerase (Promega), 0.5 μl of a 10 μM oligonucleotide mixture, containing either 1 of 54 TRAV forward primers or 1 of 65 TRBV forward primers, and 0.32 μl of the 10 μM reverse primer for the TCR constant region under the following conditions: 1) 96°C for 2 minutes, 2) 35 cycles of 96°C for 15 seconds, 57°C for 30 seconds, and 72°C for 1 minute, and 3) 72°C for 3 minutes.

### Sequencing and data analysis

One microliter of the PCR products was treated with 0.2 μl of ExoSAP-IT (usb) at 37°C for 15 minutes and subsequently at 80°C for 15 minutes. Sequencing reactions were performed in a 9 μl of reaction mixture consisting of 1 μl of the ExoSAP-IT-treated PCR products, 0.15 μl of 10 μM TCR reverse primer (hTCR-alpha-1st or hTCR-alpha-1st), 1.5 μl of 5x sequencing buffer, 1 μl of BigDye® Terminator v3.1, and 5.35 μl of water. The mixture was incubated at 96°C for 1 minutes followed by 25 cycles of 96°C for 10 seconds and 62°C for 1 minute. The sequences were determined with 3500 and 3500xL Genetic Analyzer (Applied Biosystem, USA). The repertoire of TCRα and TCRβ chains was analyzed by the IMGT/V-QUEST search tool (http://www.imgt.org/IMGT_vquest/vquest?livret=0&Option=humanTcR), and germ-line transcripts were identified by searching against the human genome sequences (BLAST search: http://blast.ncbi.nlm.nih.gov/).

The presence of dual TCRs was detected by sequence analysis with Sequence Scanner v1.0 software (Applied Biosystem, USA). Individual TCRs were amplified by PCR with a single forward primer designed for each variable segment and the TCR reverse primer (hTCR-CA-R9 for TCRα chain or hTCR-CB1-R6 for TCRβ chain). Sequencing reactions and data analysis were performed as described above. If PCR products show the sequences of two alpha and one beta chains or those of one alpha and two beta chains, a cell is evaluated to contain dual TCR.

## Supporting Information

Figure S1 **Non-canonical rearrangement of the alpha chain.**
(TIF)

Figure S2 **Conservation of CDR3α amino acid sequences created by TRAV1-2 and TRAJ31 rearrangements in early effector memory subset.** CDR3α amino acid sequences created by TRAV1-2 and TRAJ31 rearrangements were identified by IMGT/V-Quest tool, and the conservation was analyzed by Multiple Align Show (http://www.bioinformatics.org/SMS/multi_align.html). Amino acid sequences having 100% of identity and 50% of similarity are shown in black and dark gray, respectively.
(TIF)

Table S1 Nucleotide sequences of TCRα and β used in this study.
(XLSX)

Table S2 TCR usage and amino acid sequence of CDR3 region. #: Frame shift, ** Stop codon
(XLSX)

Table S3 Characterization of early effector memory cells carrying rearrangements between TRAV1-2 and TRAJ33. #: Frame shift, ** Stop codon
(DOCX)

Table S4 Characterization of TCRα/β clonotypes in CD8^+^ T-cell subsets.
(DOCX)

Table S5 Primer sequences for 5′-RACE and multiplex PCR methods for amplification of human TCRα/β chains.
(DOCX)

## References

[1] Guidotti LG, Chisari FV (1996). To kill or to cure: options in host defense against viral infection.. Curr Opin Immunol.

[2] Levy JA, Mackewicz CE, Barker E (1996). Controlling HIV pathogenesis: the role of the noncytotoxic anti-HIV response of CD8+ T cells.. Immunol Today.

[3] Robbins PF, Dudley ME, Wunderlich J, El-Gamil M, Li YF (2004). Cutting edge: persistence of transferred lymphocyte clonotypes correlates with cancer regression in patients receiving cell transfer therapy.. J Immunol.

[4] Kaech SM, Ahmed R (2001). Memory CD8+ T cell differentiation: initial antigen encounter triggers a developmental program in naive cells.. Nat Immunol.

[5] van Stipdonk MJ, Lemmens EE, Schoenberger SP (2001). Naive CTLs require a single brief period of antigenic stimulation for clonal expansion and differentiation.. Nat Immunol.

[6] Tomiyama H, Matsuda T, Takiguchi M (2002). Differentiation of human CD8(+) T cells from a memory to memory/effector phenotype.. J Immunol.

[7] Bassing CH, Swat W, Alt FW (2002). The mechanism and regulation of chromosomal V(D)J recombination.. Cell.

[8] Davis MM, Bjorkman PJ (1988). T-cell antigen receptor genes and T-cell recognition.. Nature.

[9] Gellert M (1992). Molecular analysis of V(D)J recombination.. Annu Rev Genet.

[10] Gellert M (2002). V(D)J recombination: RAG proteins, repair factors, and regulation.. Annu Rev Biochem.

[11] Jung D, Alt FW (2004). Unraveling V(D)J recombination; insights into gene regulation.. Cell.

[12] Gorski J, Yassai M, Zhu X, Kissela B, Kissella B (1994). Circulating T cell repertoire complexity in normal individuals and bone marrow recipients analyzed by CDR3 size spectratyping. Correlation with immune status.. J Immunol.

[13] Pannetier C, Cochet M, Darche S, Casrouge A, Zoller M (1993). The sizes of the CDR3 hypervariable regions of the murine T-cell receptor beta chains vary as a function of the recombined germ-line segments.. Proc Natl Acad Sci U S A.

[14] Robins HS, Campregher PV, Srivastava SK, Wacher A, Turtle CJ (2009). Comprehensive assessment of T-cell receptor beta-chain diversity in alphabeta T cells.. Blood.

[15] Freeman JD, Warren RL, Webb JR, Nelson BH, Holt RA (2009). Profiling the T-cell receptor beta-chain repertoire by massively parallel sequencing.. Genome Res.

[16] Robins HS, Srivastava SK, Campregher PV, Turtle CJ, Andriesen J (2010). Overlap and effective size of the human CD8+ T cell receptor repertoire.. Sci Transl Med.

[17] Shendure J, Ji H (2008). Next-generation DNA sequencing.. Nat Biotechnol.

[18] Sherwood AM, Desmarais C, Livingston RJ, Andriesen J, Haussler M (2011). Deep sequencing of the human TCRgamma and TCRbeta repertoires suggests that TCRbeta rearranges after alphabeta and gammadelta T cell commitment.. Sci Transl Med.

[19] Dash P, McClaren JL, Oguin TH 3rd Rothwell W, Todd B, et al (2011). Paired analysis of TCRalpha and TCRbeta chains at the single-cell level in mice.. J Clin Invest.

[20] Hamrouni A, Aublin A, Guillaume P, Maryanski JL (2003). T cell receptor gene rearrangement lineage analysis reveals clues for the origin of highly restricted antigen-specific repertoires.. J Exp Med.

[21] Ozawa T, Tajiri K, Kishi H, Muraguchi A (2008). Comprehensive analysis of the functional TCR repertoire at the single-cell level.. Biochem Biophys Res Commun.

[22] Frohman MA, Dush MK, Martin GR (1988). Rapid production of full-length cDNAs from rare transcripts: amplification using a single gene-specific oligonucleotide primer.. Proc Natl Acad Sci U S A.

[23] Edwards MC, Gibbs RA (1994). Multiplex PCR: advantages, development, and applications.. PCR Methods Appl.

[24] Takata H, Takiguchi M (2006). Three memory subsets of human CD8+ T cells differently expressing three cytolytic effector molecules.. J Immunol.

[25] Sikes ML, Gomez RJ, Song J, Oltz EM (1998). A developmental stage-specific promoter directs germline transcription of D beta J beta gene segments in precursor T lymphocytes.. J Immunol.

[26] Villey I, Quartier P, Selz F, de Villartay JP (1997). Germ-line transcription and methylation status of the TCR-J alpha locus in its accessible configuration.. Eur J Immunol.

[27] Anderson SJ, Chou HS, Loh DY (1988). A conserved sequence in the T-cell receptor beta-chain promoter region.. Proc Natl Acad Sci U S A.

[28] Deng X, Sun GR, Zheng Q, Li Y (1998). Characterization of human TCR Vbeta gene promoter. Role of the dodecamer motif in promoter activity.. J Biol Chem.

[29] Yang S, Cohen CJ, Peng PD, Zhao Y, Cassard L (2008). Development of optimal bicistronic lentiviral vectors facilitates high-level TCR gene expression and robust tumor cell recognition.. Gene Ther.

[30] Inlay M, Xu Y (2003). Epigenetic regulation of antigen receptor rearrangement.. Clin Immunol.

[31] Padovan E, Casorati G, Dellabona P, Giachino C, Lanzavecchia A (1995). Dual receptor T-cells. Implications for alloreactivity and autoimmunity.. Ann N Y Acad Sci.

[32] Singh N, Bergman Y, Cedar H, Chess A (2003). Biallelic germline transcription at the kappa immunoglobulin locus.. J Exp Med.

[33] Sleckman BP, Gorman JR, Alt FW (1996). Accessibility control of antigen-receptor variable-region gene assembly: role of cis-acting elements.. Annu Rev Immunol.

[34] Schlissel MS, Baltimore D (1989). Activation of immunoglobulin kappa gene rearrangement correlates with induction of germline kappa gene transcription.. Cell.

[35] Kobayashi Y, Tycko B, Soreng AL, Sklar J (1991). Transrearrangements between antigen receptor genes in normal human lymphoid tissues and in ataxia telangiectasia.. J Immunol.

[36] Miossec C, Faure F, Ferradini L, Roman-Roman S, Jitsukawa S (1990). Further analysis of the T cell receptor gamma/delta+ peripheral lymphocyte subset. The V delta 1 gene segment is expressed with either C alpha or C delta.. J Exp Med.

[37] Ueno T, Tomiyama H, Fujiwara M, Oka S, Takiguchi M (2003). HLA class I-restricted recognition of an HIV-derived epitope peptide by a human T cell receptor alpha chain having a Vdelta1 variable segment.. Eur J Immunol.

