# Supplementary figures and images for: Unbiased Analysis of TCRα/β Chains at the Single-Cell Level in Human CD8+ T-Cell Subsets
Source: PLoS One. 2012 Jul 6;7(7):e40386. doi: 10.1371/journal.pone.0040386 (PMC3391256; doi:10.1371/journal.pone.0040386)

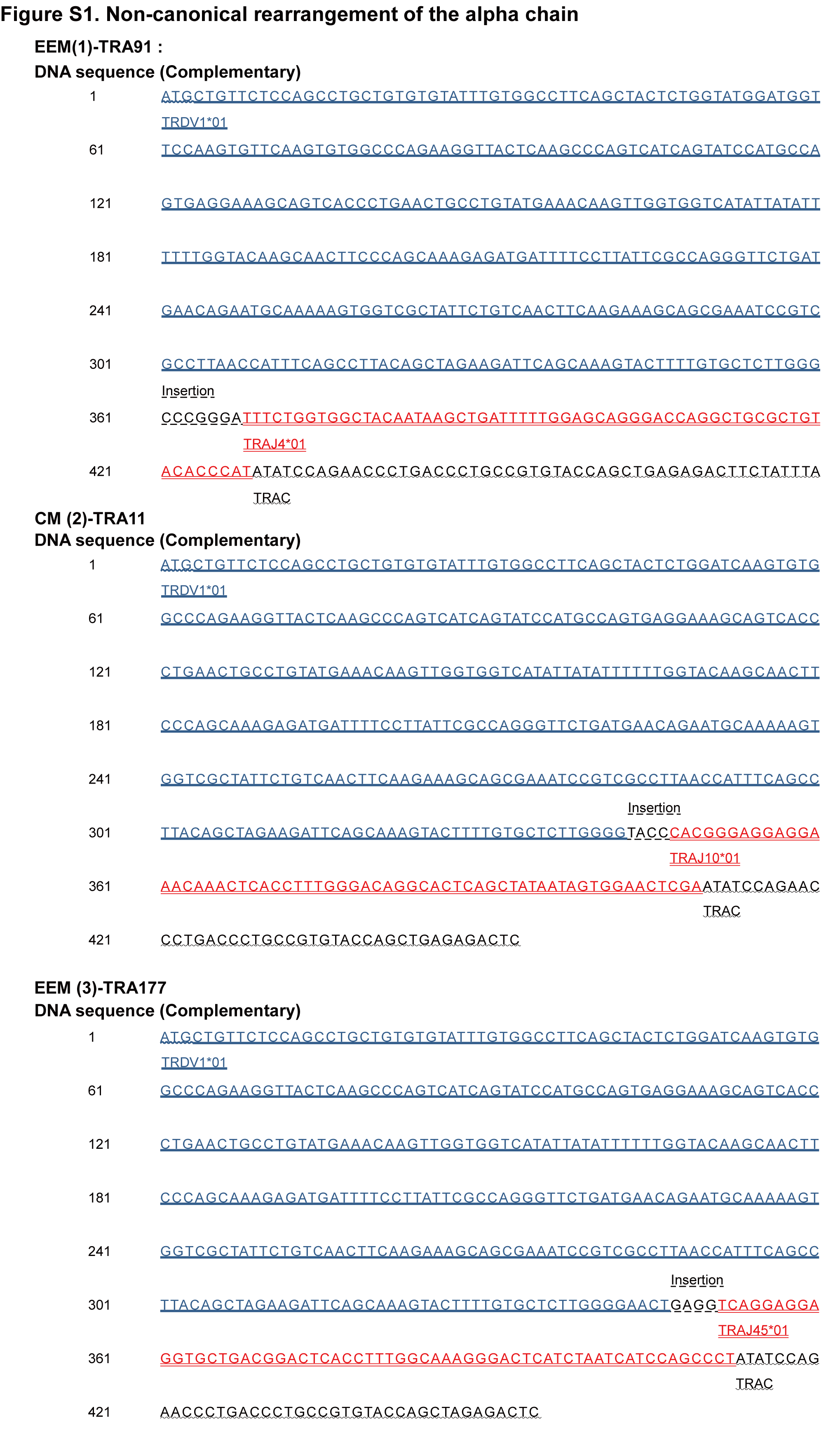

Supplement: Figure S1 — Non-canonical rearrangement of the alpha chain. (TIF) [file pone.0040386.s001.tif]

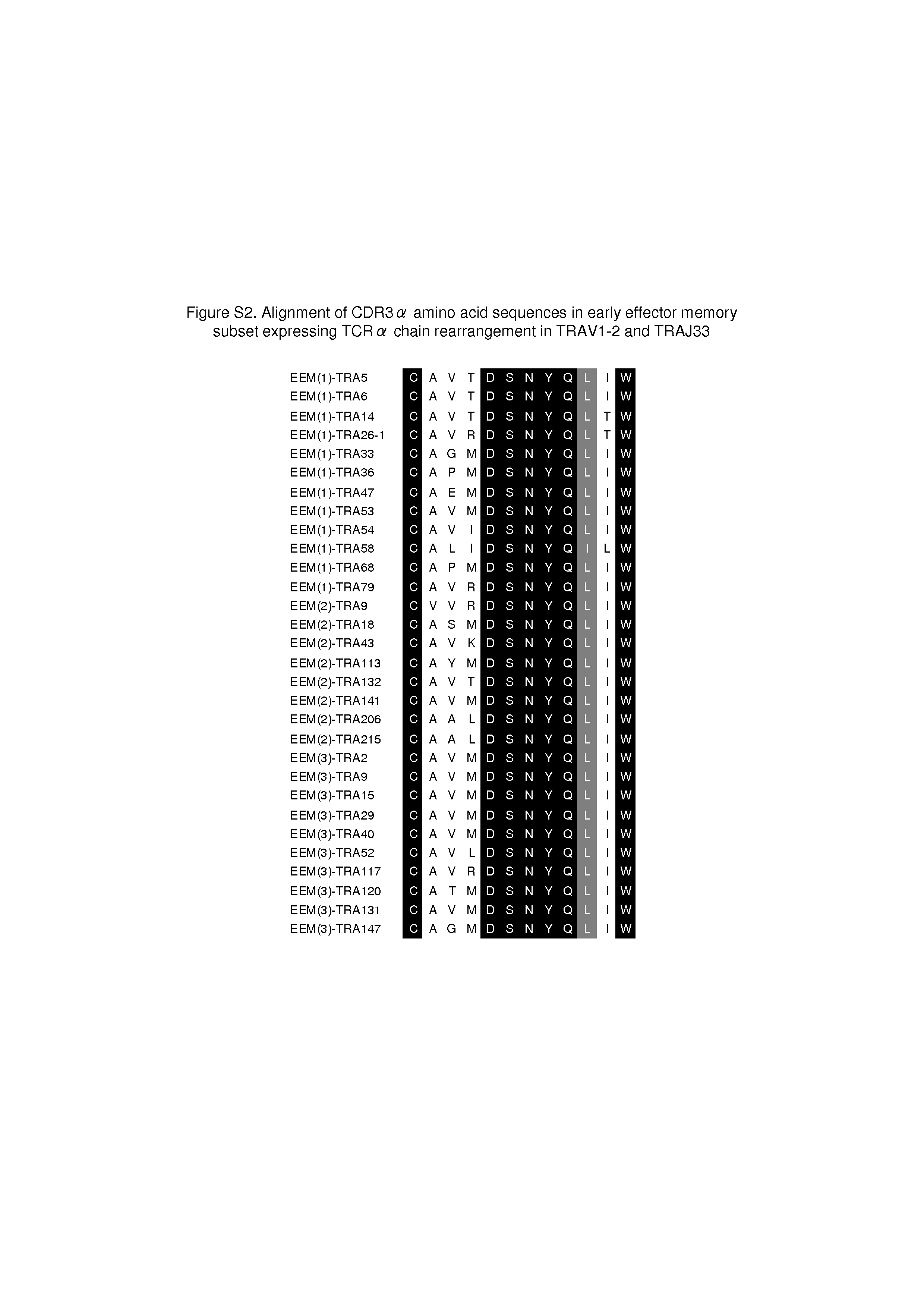

Supplement: Figure S2 — Conservation of CDR3α amino acid sequences created by TRAV1-2 and TRAJ31 rearrangements in early effector memory subset. CDR3α amino acid sequences created by TRAV1-2 and TRAJ31 rearrangements were identified by IMGT/V-Quest tool, and the conservation was analyzed by Multiple Align Show (http://www.bioinformatics.org/SMS/multi_align.html). Amino acid sequences having 100% of identity and 50% of similarity are shown in black and dark gray, respectively. (TIF) [file pone.0040386.s002.tif]
