# Supplementary material for: Unbiased Analysis of TCRα/β Chains at the Single-Cell Level in Human CD8+ T-Cell Subsets
Source: PLoS One. 2012 Jul 6;7(7):e40386. doi: 10.1371/journal.pone.0040386 (PMC3391256; doi:10.1371/journal.pone.0040386)
Supplement: Table S3 — Characterization of early effector memory cells carrying rearrangements between TRAV1-2 and TRAJ33. #: Frame shift, ** Stop codon (DOCX) [file pone.0040386.s005.docx]

**Table S3. Characterization of early effector memory cells carrying rearrangements between TRAV1-2 and TRAJ33**

N.D: Not detected
