# Supplementary material for: Unbiased Analysis of TCRα/β Chains at the Single-Cell Level in Human CD8+ T-Cell Subsets
Source: PLoS One. 2012 Jul 6;7(7):e40386. doi: 10.1371/journal.pone.0040386 (PMC3391256; doi:10.1371/journal.pone.0040386)
Supplement: Table S4 — Characterization of TCRα/β clonotypes in CD8+ T-cell subsets. (DOCX) [file pone.0040386.s006.docx]

**Table S4. Characterization of TCRα/β clonotypes in CD8^+^ T-cell subsets**

#: Frame shift, N: Naive, CM: Central memory, EEM: Early effector memory, LEM: Late effector memory, E: Effector
