# Supplementary material for: Unbiased Analysis of TCRα/β Chains at the Single-Cell Level in Human CD8+ T-Cell Subsets
Source: PLoS One. 2012 Jul 6;7(7):e40386. doi: 10.1371/journal.pone.0040386 (PMC3391256; doi:10.1371/journal.pone.0040386)
Supplement: Table S5 — Primer sequences for 5′-RACE and multiplex PCR methods for amplification of human TCRα/β chains. (DOCX) [file pone.0040386.s007.docx]

**Table S5. Primer sequences for 5’-RACE and multiplex PCR methods for amplification of human TCRα/β chains**
